# Building trust and privacy in cross-border health data sharing for European cancer research

**DOI:** 10.1093/radadv/umag018

**Published:** 2026-03-30

**Authors:** Ricard Martínez Martínez, Ana de Marco, Ignacio Blanquer, Luis Martí-Bonmatí

**Affiliations:** Instituto Universitario de Investigación de Robótica y Tecnologías de la Información y Comunicación. Universitat de València , Valencia, 46980, Spain; Biomedical Imaging Research Group, Instituto de Investigación Sanitaria La Fe, Valencia, 46026, Spain; Instituto de Instrumentación Para la Imagen, Universitat Politècnica de València, Valencia, 46022, Spain; Biomedical Imaging Research Group, Instituto de Investigación Sanitaria La Fe, Valencia, 46026, Spain; Radiology Department, Hospital Universitario y Politécnico La Fe, Valencia, 46026, Spain

**Keywords:** European Health Data Space, GDPR, data governance, anonymization, federated learning, cancer imaging

## Abstract

Data-driven research using artificial intelligence (AI) is transforming biomedical science, yet its application in medical imaging remains limited by fragmented datasets, heterogeneous legislation, and ethical uncertainties. The European Cancer Imaging Initiative (EUCAIM) addresses these barriers by establishing a federated, secure and interoperable European imaging infrastructure, fostering a trusted ecosystem for AI-enabled research. EUCAIM brings privacy, ethics, and security within a single, coherent operational framework. The project implements a risk-based, compliance-by-default approach that embeds Data Protection Impact Assessments throughout system design, translating legal requirements into verifiable technical safeguards. Its “de facto” anonymization model, aligned with the General Data Protection Regulation and Court of Justice jurisprudence, combines multi-stage anonymization pipelines, cryptographic hashing, and automated re-identification-risk analyses to deliver a federated Secure Processing Environment (SPE) for researchers. This federated infrastructure is consistent with the European Health Data Space Regulation (EHDSR) and national security frameworks, and ensures data sovereignty, interoperability, and accountability. A comprehensive governance and contractual framework, including Data Sharing and Transfer Agreements, clearly delineates roles and responsibilities, while the Data Access Committee provides robust ethical oversight. EUCAIM thus offers a lawful, secure, and sustainable model of a federated secure environment for the reuse of imaging data, advancing a genuinely data-driven research ecosystem.

**Abbreviations** 

AI - Artificial Intelligence DPA - Data Protection Authority DPIA - Data Protection Impact Assessment DSA - Data Sharing Agreement DTA - Data Transfer Agreement EUCAIM - European Cancer Imaging Initiative EHDSR - European Health Data Space Regulation GDPR - General Data Protection Regulation SPE - Secure Processing Environment • • •

**Summary** 

EUCAIM provides a blueprint for secure, ethical, and sustainable reuse of cancer imaging across Europe, integrating regulation, technology, and clinical research under GDPR-based data privacy and control.

**Essentials** 

- Synchronized legal, ethical, and technical frameworks are essential for trustworthy reuse of health data.
- EUCAIM integrates de facto anonymization, federated processing on secure environments, and standardized agreements to ensure GDPR-compliant research.
- A multi-layered governance model connects legal accountability, ethical oversight, and a secure technical infrastructure.
- The EUCAIM approach demonstrates that compliance and innovation can coexist, providing a blueprint for the privacy and security requirements of future European Health Data Spaces.

## Introduction

**Figure 1:**
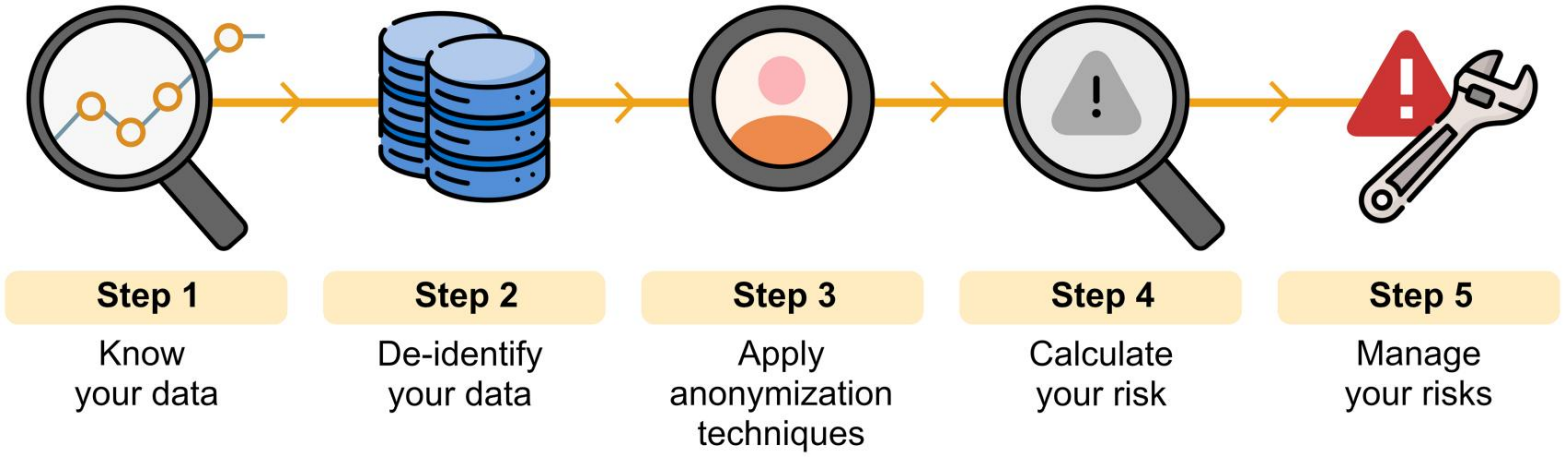
Steps for anonymization. *Source: Agencia Española de Protección de Datos—*(Spanish Data Protection Agency). Guide to basic anonymization. Prepared by the National Data Protection Authority of Singapore (PDPC—Personal Data Protection Commission Singapore).

This review focuses on the essential infrastructure that enables practical and trustworthy data-driven research in medical imaging, particularly in cross-border settings. Over the past decade, the concept of real-world data has gained increasing strength and legitimacy.^1^ Large datasets, sourced from single hospitals, multicenter studies, or multi-country projects, are essential, and they must be managed in a secure, reliable environment to ensure that data-driven research using artificial intelligence (AI) is precise, reproducible, and safe (Figure 1).

### Challenges to cross-border health data sharing for researchers

Unfortunately, across the European Union, managing medical imaging and related health data for secondary use has encountered ethical and legal barriers that have proven extremely difficult to overcome. Despite the sustained efforts of the scientific community, the regulatory framework established by the General Data Protection Regulation (GDPR)^2^ has not provided the necessary conditions for lawful and efficient access to large-scale datasets.^3^ Researchers repeatedly face legal processes that are excessively complex and time-consuming, provide limited legal certainty, and too often operate under an implicit presumption of wrongdoing.

The greatest difficulty has stemmed from the widely accepted idea that large-scale data processing is feasible only through anonymization to preserve patients’ privacy. Yet anonymization has proven particularly challenging, largely due to the restrictive stance of many Data Protection Authorities (DPAs), whose interpretation often emphasizes the practical impossibility of fully anonymizing health data rather than identifying legally sound and practically viable pathways for responsible data use.^4^

As a result, researchers have increasingly relied on pseudonymization techniques, which provide strong guarantees of privacy and data security.^5^ Nevertheless, in most jurisdictions -and with very limited exceptions, such as under Spanish legislation—the use of pseudonymized data for research purposes remains conditional on obtaining the data subject’s explicit informed consent. Furthermore, the GDPR grants Member States the authority to define specific rules governing the processing of health data for research purposes, thereby adding further layers of regulatory fragmentation.

These factors have imposed severe constraints on scientific progress. In practice, many research datasets are built for statistical representativeness rather than as truly large-scale repositories capable of supporting AI-driven methods. The heavy regulatory burden fosters informational silos anchored to national law, while research capacity is often constrained by internal institutional rules and regulations, health-system policies, and defensive stances of ethics committees and Data Protection Officers. Finally, regulatory asymmetries among Member States remain a major barrier to the development of large pan-European research consortia.

The European Cancer Imaging (EUCAIM) initiative represents a paradigm shift. Rather than focusing on policy analysis alone, this infrastructure provides an implementation-level example of how federated access, anonymization strategies, governance frameworks, and SPEs translate regulatory principles into practical research workflows. By federating fragmented imaging repositories across Europe, EUCAIM^6^ combines legal compliance, technical excellence, and ethical accountability.

### Alternative frameworks for cross-institutional data sharing

To situate EUCAIM within a global landscape, its approach can be compared with other major international infrastructures that enable secondary use of health data. In the United Kingdom, Trusted Research Environments (TREs),^7^ such as National Health Service Digital’s Secure Data Environment, provide controlled access to health data within a national regulatory framework, and are built around the strict privacy and security principle that “data stay where they are,” an approach that conceptually aligns with the EUCAIM’s federated model.

However, TREs operate within a single jurisdiction, whereas EUCAIM must enable interoperability and trust between 27 Member States under the GDPR. Moreover, TRE frameworks rely on de-identification and controlled access to pseudonymized data. By contrast, EUCAIM adopts a de facto anonymization strategy aimed at minimizing re-identification risk: both direct and indirect identifiers are removed from imaging data, while alphanumeric data are de-identified at the data holder level through secure hashing of sensitive information.

In the United States, the National Institute of Health’s (NIH) Data Commons initiative promotes Findable, Accessible, Interoperable, Reusable principles, and cloud-based data sharing. It operates under a governance model centered on informed participant consent and oversight by individual Institutional Review Boards, rather than a rights-based regulatory framework such as the GDPR. EUCAIM distinguishes itself by explicitly embedding regulatory compliance into its technical architecture, providing standardized governance mechanisms designed for cross-border scalability, and aligning its model with the current European Health Data Space Regulation (EHDSR).

The Data Governance Act defines a SPE as “the physical or virtual environment and organizational means to ensure compliance with Union law, such as Regulation (EU) 2016/679, in particular with regard to data subjects’ rights, intellectual property rights, and commercial and statistical confidentiality, integrity and accessibility, as well as with applicable national law, and to allow the entity providing the secure processing environment to determine and supervise all data processing actions, including the display, storage, download and export of data and the calculation of derivative data through computational algorithms.”

The EHDSR defines the measures that a SPE should feature:

- Restrict access to authorized people listed in the respective data permit.
- Minimize the risk of the unauthorized reading, copying, modification or removal of electronic health data hosted in the SPE through state-of-the-art technological means.
- Limit the input of electronic health data and the inspection, modification or deletion of electronic health data hosted in the SPE to a limited number of authorized identifiable individuals.
- Ensure that data users have access only to the electronic health data covered by their data permit, by means of individual and unique user identities and confidential access modes only.
- Keep identifiable logs of access to the SPE for the period of time necessary to verify and audit all processing operations in that environment.
- Ensure compliance and monitor the security measures referred to in this Article to mitigate potential security threats.

## Methods

This manuscript presents a narrative, implementation-oriented review that combines elements of policy analysis, legal interpretation, and infrastructure description. Unlike systematic or scoping reviews that aim to comprehensively synthesize comparative evidence, this practice-based, experience-driven framework review focuses on translating regulatory principles into operational research workflows. The analysis is grounded in the authors’ direct involvement in the design, deployment, and operation of the EUCAIM infrastructure, including the development of governance templates, privacy risk-assessment methodologies, anonymization pipelines, onboarding procedures for data holders, and Secure Processing Environment (SPE) configurations. Thus, it is intended to serve as a practical reference model rather than an exhaustive synthesis of the literature.

EUCAIM’s overarching goal is to provide the scientific community with a secure ecosystem of health data, equipped with high-capacity processing capabilities, advanced analytical tools for the development and validation of AI models. Within the EUCAIM platform there are: 83 datasets with 106 968 subjects, summing up more than 10 million individual images, 57 software tools registered in the catalogue, a hyperontology with over 3300 standard terms for 22 cancer types, a dedicated processing node with over 1000 cores and 25 GPUs, 203 registered users from 16 countries, 167 data holders showing interest to be linked from 29 countries.

**Figure 2:**
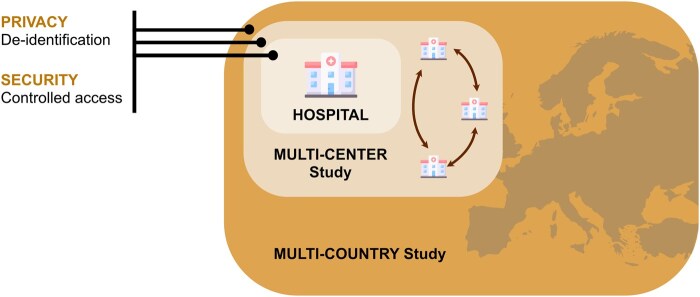
Balancing privacy, security, and scientific value in the secondary use of data is a central challenge. EUCAIM’s multi-layered approach—combining technical safeguards, legal agreements, and governance oversight—enables data sharing that meets both regulatory requirements and scientific needs, demonstrating that privacy and research value are not mutually exclusive when appropriate controls are in place.

The EUCAIM initiative operates within a dynamic regulatory environment. Building on experience from the AI for Health Imaging projects, such as CHAIMELEON^8^ and PRIMAGE,^9^ EUCAIM’s methods are shaped by practical lessons on how regulatory requirements can be embedded directly into technical workflows that can be replicated by individual radiology departments and research units. In practice, this means that EUCAIM’s approach focuses on operational uniformity across sites, including common documentation templates, harmonized dataset descriptors, unified onboarding workflows for data holders, and early incorporation of requirements that will facilitate future interoperability with the EHDSR.^10^ These elements are directly relevant to multicenter and multinational imaging studies, as well as AI validation pipelines (Figure 2).

**Figure 3:**
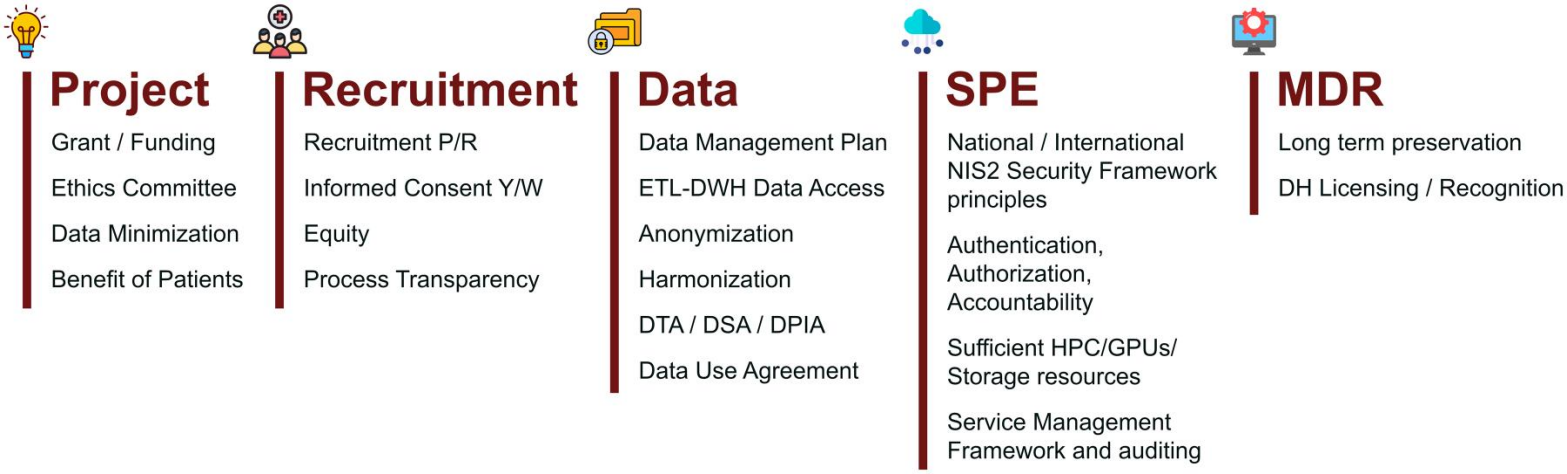
Data and documentation workflow across European health research projects, ensuring auditability. The workflow traces data from source institutions through anonymization, contractual agreements (DSAs, DTAs), and governance review by the Data Access Committee to the Secure Processing Environment. Key decision points include DPIA assessment, tier classification (1-3), and data-access approval. DH, Data Holder; DPIA, Data Protection Impact Assessment; DSA, Data Sharing Agreement; DTA, Data Transfer Agreement; DWH, Data Warehouse; ETL, Extract, Transform, and Load; MDE, Metadata Extraction; P/R, Prospective/Retrospective; Y/W, Yes/Waive; SPE, Secure Processing Environment; TRE, Trusted Research Environment.

To meet these objectives, EUCAIM has implemented a multi-layered methodological strategy, described below, which combines functional and legal mechanisms and documentation to guarantee compliance, flexibility, and scientific usability across the consortium (Figure 3). This has been deliberately designed to enable imaging researchers and data scientists to understand how regulatory conditions translate into specific technical features of the platform, including dataset harmonization workflows, federated access governance, and standardized documentation packages.

### Risk-based data protection assessment and mitigation

The development and deployment of EUCAIM requires full application of the mechanisms established under the GDPR. In radiological research, the priority is to ensure that image reuse and data workflows are technically configured to preserve patient privacy while enabling researchers to perform clinically meaningful analyses. This operational perspective is essential as radiology users require clarity on how privacy safeguards translate into day-to-day data access and algorithm development. Data Protection Impact Assessments (DPIAs), provided for in Article 35 of the GDPR, serve as tools for evaluating whether a dataset or processing workflow is suitable for integration into a federated research environment. They guide the implementation of key safeguards, including access-segregated workspaces, traceability controls, and appropriate levels of data minimization. Every technological component, AI algorithm, or multicenter study integrated into EUCAIM undergoes a DPIA, with a focus on how the assessments enable concrete operational decisions. This includes determining if a study can run on fully anonymized data or must apply constraints on exportability or model inspection. For researchers, this approach makes the regulatory compliance much clearer, since it directly shapes the architecture through which data is used.

### De facto data anonymization: Dual strategy on anonymization

**Figure 4:**
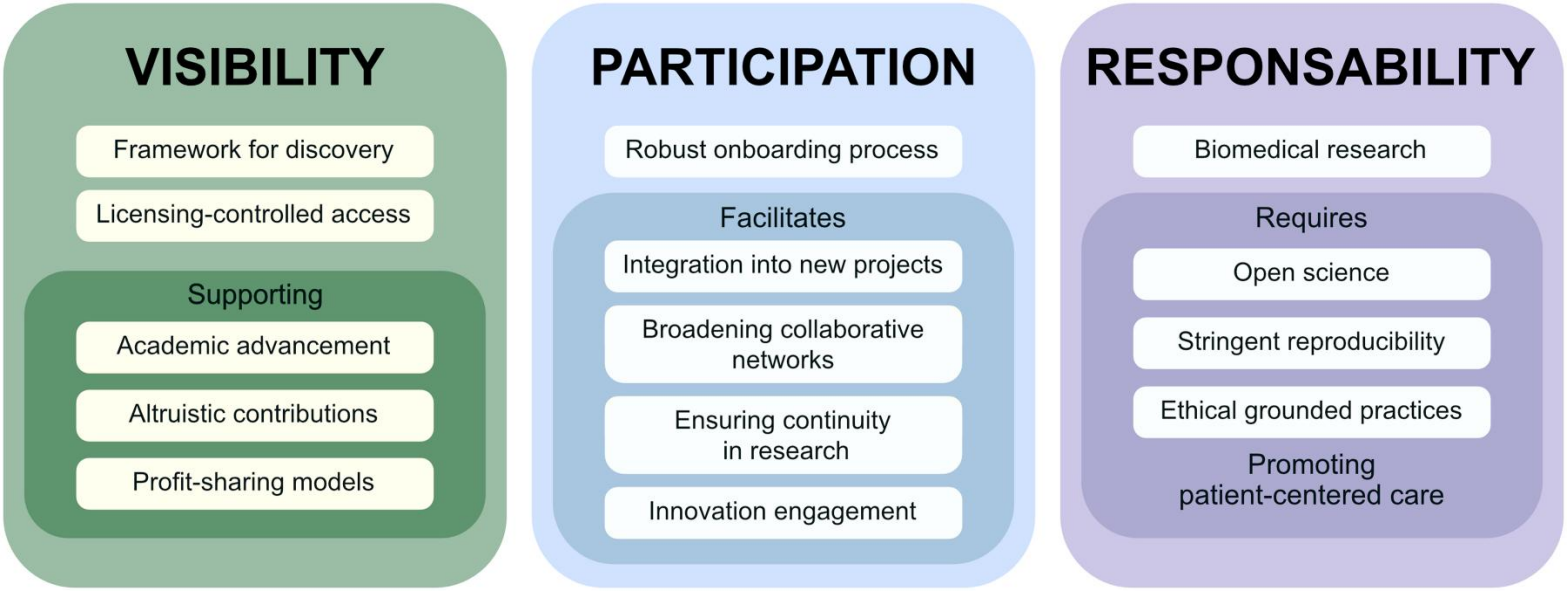
The value of health data sharing: visibility, participation, and responsibility. The societal benefits of trustworthy health data sharing and shared responsibility among stakeholders, including data holders, researchers, regulators, and patients, are highlighted. The framework positions data sharing as both a technical challenge and a collective endeavor in the healthcare service.

In the field of anonymization, EUCAIM has been designed to implement processes consistent with the provisions of Recital 26 of the GDPR and aligned with the position of the Court of Justice of the European Union, including the position-dependent understanding of identifiability. This remains controversial and inevitably carries some risks. Nevertheless, the ecosystem described below operates in accordance with EHDSR rules governing the processing of pseudonymized data without requiring explicit participant consent (Figure 4).

EUCAIM’s strategy follows several methodological frameworks issued by different DPAs,^11–13^ ensuring alignment with the privacy-protection state of the art and adopting a risk-based orientation.

To clarify the distinction between “de facto anonymization” and traditional pseudonymization, consider the following examples. Under traditional pseudonymization, researchers may access datasets in which patient names are replaced with codes, for example, Patient001, Patient002-, while the original institution retains the key linking codes to identities. The data, therefore, remains personal data, and processing requires either informed consent or another valid legal basis. Identifiability persists because the means of reidentification continues to exist, even if technically separated. In contrast, under EUCAIM’s de facto anonymization model, a combination of multiple technical and organizational measures renders re-identification effectively impossible for any party accessing data within the SPE. This is achieved through a dual-strategy architecture that combines local anonymization by Data Holders and environment-level safeguards within the SPE. Direct identifiers are removed at source; dates are shifted or generalized; DICOM metadata are stripped; domain-specific sanitization, such as defacing, is applied where applicable; cryptographic hashing prevents linkage to external records; and researchers access data exclusively through controlled interfaces that prohibit data export. Strong authentication, role-based access control, comprehensive logging, and output-disclosure controls further limit re-identification risk during data use. The key distinction is that while absolute anonymization may be theoretically impossible, EUCAIM achieves a state in which re-identification requires “disproportionate effort” (per Recital 26 GDPR), considering the technical, organizational and legal constraints on authorized users. Such data may be treated as anonymous for practical and legal purposes, in order with the EHDSR framework, which permits the processing of pseudonymized data within secure environments without explicit consent.

The anonymization process applies to both imaging and clinical data. For medical images, anonymization is performed locally by the Data Holders, removing all direct identifiers and deleting or generalizing all metadata that may entail a re-identification risk, such as dates, geographic coordinates, or device-specific information. Regarding clinical data, anonymization is also carried out in phases, combining identifier removal, pseudonymization, and minimization of quasi-identifiers to strengthen privacy protection.

Integration of imaging and clinical data is performed locally under the control of each Data Holder, using cryptographic hashing techniques^14^ that enable dynamic linkage between datasets while preventing visibility by EUCAIM or end users. Both during local harmonization and prior to integration into the federated platform, re-identification risk analyses are conducted using dedicated software. These analyses allow datasets that fail to meet the required anonymization thresholds to be rejected while determining when pseudonymization is sufficient for controlled processing within the secure environment defined by the EHDSR.

### A federated and secure data processing environment

EUCAIM is structured as a closed, federated and safe ecosystem in accordance with EHDSR specifications and aligned with the Spanish National Security Framework,^15^ which governs information systems within public administrations. This configuration serves as the operational layer, ensuring that all processing activities occur within a framework of legal certainty, security, and ethical accountability, supported by traceability and auditability mechanisms. EUCAIM promotes data sharing to enhance institutional visibility, participation, and accountability in the development of imaging biomarkers and precision medicine (Figure 4).

The project implements a federated architecture designed to support real research workflows. When integrating nodes, 3 progressive levels of trustworthiness are defined^16^:

- Tier 1 (Non-aligned): Data collections outside the scope of federated integration.
- Tier 2 (Partial alignment): Legal and technical requirements are largely satisfied, enabling federated discovery and querying.
- Tier 3 (Full alignment and SPE ready): Data providers meet advanced legal, ethical, and technical criteria, enabling distributed processing within SPEs.

Each federated node must comply with established standards for interoperability, encryption, and access control, while retaining full data sovereignty at the source. This tiered approach allows institutions to participate early while progressively increasing their level of technical and governance maturity.

All processes are governed by an integrated framework combining technical, ethical, and legal oversight to ensure security and compliance. For Tier 3 participation and SPE-enabled processing, EUCAIM operationalizes EHDSR requirements through technically enforced controls, aligned with the requirements of the SPEs:

- Restricted access to data to strongly authenticated users who host a data permit that identifies the accessible datasets.
- Access to data exclusively through network-restricted and isolated virtual research environments deployed on the SPEs.
- Detailed auditing and monitoring of users’ actions.
- Audited catalogue of applications deployed in the SPEs.
- Encrypted storage with data minimization policies to limit the risk of privacy leaks.
- Audited retrieval of the output produced by the researchers.

For researchers and radiologists, this enables data analysis at the point of generation, validation of algorithms across heterogeneous cohorts without data leaving individual healthcare systems, and cross-border collaboration that is operationally feasible rather than merely a policy aspiration.

### A comprehensive legal framework

A substantial component of EUCAIM’s legal architecture is the development of contractual instruments designed to ensure accountability. At present, the EUCAIM research consortium has a complex legal configuration, as it lacks a legal personality distinct from that of its partner institutions. Within the GDPR, this requires the use of specific instruments to regulate data flows. Accordingly, once the relevant roles—data controller, processor, and joint controller^17^—are defined, appropriate contractual arrangements are formalized through data-processing or joint-controllership agreements, as provided for in the Regulation.

Data reuse in a federated environment entails further obligations beyond data protection, including safeguards for intellectual property and information security, data cataloguing, service-level agreements, and compliance with conditions or limits set by national law. Because datasets reused within EUCAIM are anonymized, the applicable agreements differ from standard GDPR-based contracts. Accordingly, traditional data transfer agreements (DTAs) have evolved into 2 complementary instruments: a Data Sharing Agreement (DSA), governing federated data-processing scenarios, and a DTA, regulating transfers to the central node. Pending full implementation of the EHDSR, a clear operational mandate for EUCAIM is required to authorize data-access requests and ensure predictable and traceable decision making.

For Data Users, 2 mechanisms are mandatory. First, the legal representative of each participating institution must formally accept the platform’s terms and conditions.^18^^,^^19^ Second, each authorized user must sign a binding commitment ensuring confidentiality, prohibiting re-identification, and acknowledging individual responsibilities for data security. Together, these instruments establish an auditable chain of responsibility among EUCAIM, participating institutions, and individual researchers, strengthening trust within an implementation-oriented governance model.

### Governance framework

EUCAIM’s long-term sustainability requires a clearly defined organizational governance model capable of evolving alongside the infrastructure, with 2 core dimensions warranting particular attention.

First, the distributed deployment of activities across multiple partners inevitably increases organizational complexity. Beyond traditional governance functions—such as general management, boards, compliance units, and IT services—the organization must incorporate roles now standard in the data domain, such as Chief Data Officer, Chief Information Security Officer, and Data Protection Officer. A high degree of specialization among data analysts will also be required, alongside emerging profiles in AI ethics. Training is essential not only to implement data protection by design but also to meet the AI literacy objectives set out in Article 4 of the AI Act.^20^ Experience to date within EUCAIM indicates that radiologists, researchers, innovators, and technical leaders are increasingly sharing governance responsibilities and embedding regulatory requirements into established workflows.

Second, a robust governance framework must regulate both the integration of datasets and data-access authorization. This responsibility is assigned to a dedicated Data Access Committee, supported by experts in medical imaging, data protection, and AI ethics. The Data Access Committee is responsible for verifying that both institutions and individual applicants demonstrate the required ethical, legal, and technical trustworthiness, and for guiding EUCAIM’s decision-making bodies. In parallel, mechanisms for engagement with the broader community—including patients, healthcare professionals, researchers, industry, and public authorities—are developed through transparency measures and advisory councils.

## Results

Building upon a multilevel risk-based, compliance-by-design framework, EUCAIM delivers a secure, reliable, and interoperable infrastructure that enables lawful and ethically sound reuse of medical imaging data across Europe. The integration of DPIAs into system design ensures that legal obligations translate into concrete technical compliance safeguards. The deployment of a multistage anonymization pipeline, supported by cryptographic hashing and automated re-identification-risk analyses, creates a robust foundation for data protection and privacy preservation.

### Data protection impact assessment and data protection by design and by default

The DPIA provides a comprehensive pragmatic structure for risk management that extends directly into the system’s implementation. Through these data protection mechanisms, the infrastructure evolves toward what can be described as compliance by default, a practical necessity in light of the increasing complexity of the European regulatory landscape.

While data sharing enhances reproducibility, it also introduces new risks of re-identification, particularly through AI-based reconstruction techniques. Achieving a workable balance between data utility and patient protection is, therefore, essential.^21^ Within EUCAIM, this balance is addressed by operational accountability as a design principle at every stage of technological development, rather than as a post hoc correction measure.

### De facto anonymization within a secure processing environment

The creation of a closed, governed, and fully traceable processing environment mitigates many of the risks traditionally associated with open data models, in which control is lost once data are downloaded, and re-identification risks proliferate. This design not only reinforces the reliability and robustness of EUCAIM’s anonymization strategy but also operationalizes the concept of de facto anonymization. Users are restricted to executing authorized processes within the secure environment, thereby preventing unlawful access, re-identification, and data misuse.

Consequently, EUCAIM meets the core GDPR requirements, since any attempt to re-identify a patient would require disproportionate effort, breach of security safeguards, and constitute an illegal act. Moreover, the architecture structurally prevents re-identification by third parties lacking authorized access to the environment.

The federated data-processing model further guarantees that each node and its corresponding Data Holder retain full control over processing activities involving their data. This approach provides traceability, enhances transparency, and incentivizes proactive prevention of potential misuse.

The overall design follows a dual-layer approach. First, authorized users are provided with validated and user-friendly access to explore the catalogue of available datasets and processing options through the application dashboard and orchestration services. Once a data-access request is approved, the platform’s legal and technical verification, control, and traceability mechanisms enable Data Holders to maintain effective oversight of both the executed processes and the data involved.

### Anticipated benefits of the contractual and governance framework

EUCAIM has established a reference framework designed to build strong public trust in the responsible operation of a high-performance environment for data-driven and AI-enabled research. Together, the DSAs, DTAs, and Terms and Conditions constitute a comprehensive contractual framework governing every stage of the data lifecycle—from generation and processing to eventual deletion. This framework guarantees strict legal compliance and defines, with exceptional granularity, the roles, responsibilities, and obligations of all parties involved.

The implementation of governance processes through the Data Access Committee already provides valuable insights for the future deployment of the EHDSR. The evolving regulatory environment is expected to introduce new actors into the data-reuse ecosystem, as open-access organizations and initiatives expand inclusivity and global participation in health data research.

**Figure 5:**
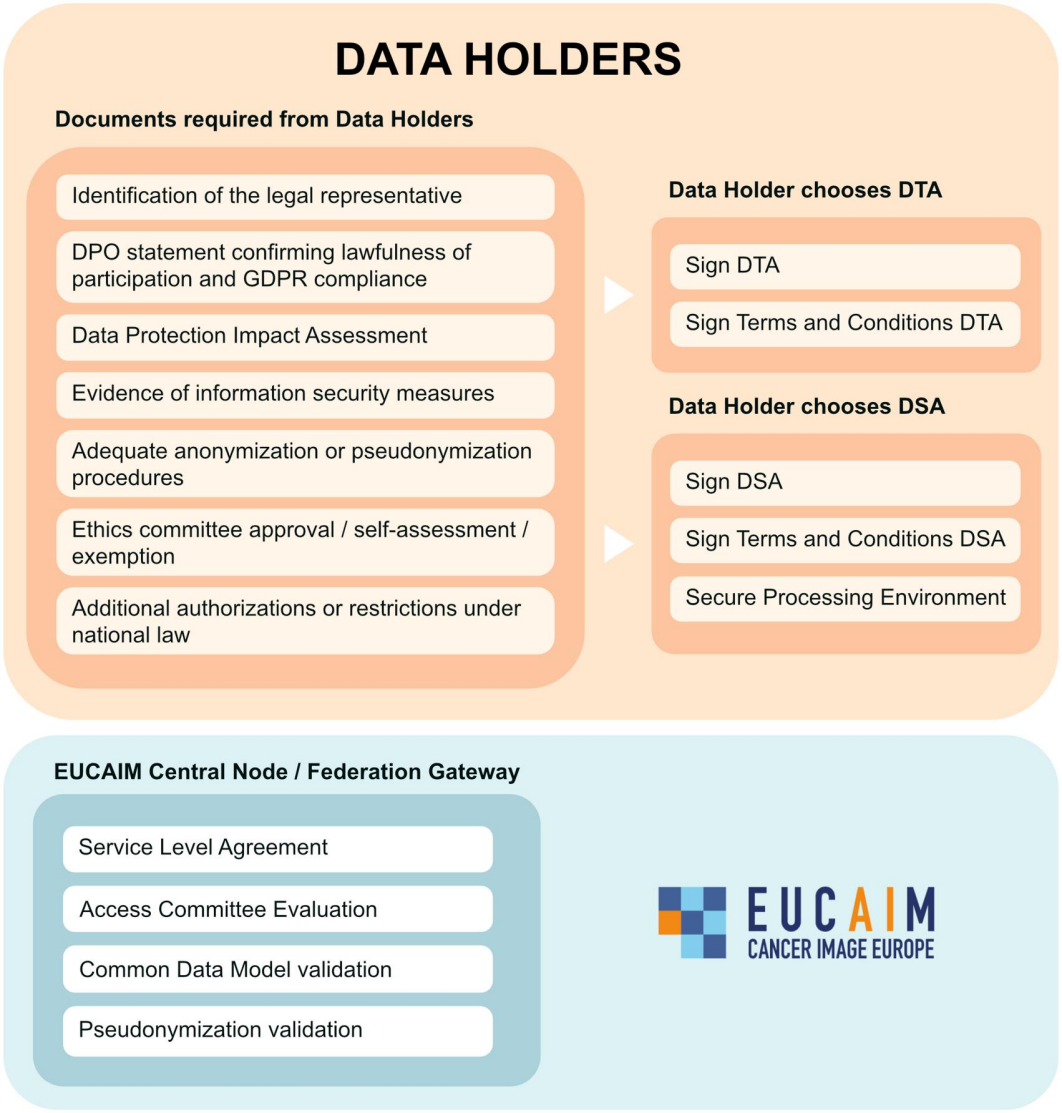
Documentation and procedures required by Data Holders. This figure describes the options for data holders to participate in EUCAIM. Whether it is as a federated node or by data collection transfer.

If the objective of dismantling data silos is achieved, new research avenues will emerge from collaborations with economics, engineering, and social sciences. In parallel, beyond traditional research and educational institutions, a new wave of innovative companies is entering the health domain. Lessons learned through current Data Access Committee procedures will serve as guidance for designing functional governance models under the EHDSR (Figure 5).

Beyond data protection, recent analyses emphasize that ethical data mining must address algorithmic bias, fairness, and accountability, and that technical privacy safeguards should incorporate mechanisms for auditability and transparency. The growing diversity of stakeholders, the proliferation of innovative initiatives requiring governance, and the need to secure the confidence not only of patients but of society at large represent a core commitment for EUCAIM.

### Limitations

Several limitations warrant acknowledgment. First, EUCAIM’s de facto anonymization model, while legally defensible, has not yet been formally endorsed by all relevant DPAs. Some DPAs remain skeptical of any framework asserting anonymization of medical imaging data. Continued engagement and dialogue with regulatory bodies will therefore be essential to achieve broader acceptance, and in the short term, divergent interpretations may slow the onboarding of new data holders.

Second, scalability remains an open challenge, particularly in terms of cost and expertise. As the platform expands beyond the current 167 data holders, demands on technical infrastructure, governance coordination, and operational oversight are expected to increase substantially, including greater requirements for computing capacity, secure storage, and specialized personnel at both central and local levels.

Third, national variations in GDPR implementation introduce additional constraints, as some Member States impose supplementary requirements—such as new ethics committee review or national security considerations—that may limit full interoperability. For individual institutions, navigating these layered approval processes can result in significant delays before datasets become available for federation.

Fourth, although comprehensive, the contractual framework imposes a substantial administrative burden on participating institutions, potentially disadvantaging smaller research centers with limited legal resources. In the short term, this may result in slower participation by less-resourced hospitals despite technical readiness.

Fifth, significant short-term technical challenges persist at the institutional level, including heterogeneity of PACS and clinical information systems, variability in data quality and metadata completeness, and limited local IT capacity to support anonymization, harmonization and secure node deployment.

Sixth, the operation of an SPE requires significant specialized human resources to fulfil the commitment of EUCAIM’s Service Level Agreements. It is important to understand that federated processing and incident detection are affected by the weakest link in the chain.

Also, organizational and cultural factors may affect early implementation, including conservative attitudes toward secondary data use, limited familiarity with federated research models, and concerns about liability or reputational risk among institutional leadership.

Finally, although EUCAIM principles are designed to be generalizable, their direct applicability outside the EU regulatory context—where the GDPR does not apply—has not yet been tested.

## Discussion

The development and deployment of the EUCAIM framework underscore the value of early integration of DPIAs, not only to streamline subsequent validation processes but also to foster shared understanding among legal, ethical, and technical teams. This early alignment has been critical in avoiding late-stage redesign of technical components and in reducing uncertainty during dataset onboarding and study authorization. In parallel, preserving sufficient data utility for imaging research has required iterative testing with clinicians, data scientists, innovators, and researchers to ensure that privacy safeguards do not compromise analytic value.

Several concrete use cases illustrate EUCAIM’s practical value for the radiology community. First, in multicenter AI model validation, researchers can train machine learning models on local data and subsequently validate them across geographically distributed datasets within the SPE. This approach enables assessment of algorithms’ generalizability across populations, imaging equipment, and clinical protocols without centralizing sensitive patient data, which is particularly relevant for evaluating model robustness prior to clinical translation.

Second, in rare disease research, federating imaging repositories across 29 countries enables EUCAIM to support cohort assembly for conditions in which no single institution holds sufficient cases, such as rare pediatric cancers or uncommon tumor subtypes, thereby enabling statistically meaningful analyses that would otherwise be infeasible.

Third, regarding regulatory-grade evidence generation, the standardized governance and documentation frameworks position EUCAIM datasets for potential use in medical device regulatory submissions, where data provenance, quality assurance, and traceability documentation of data lineage and processing steps are essential. Ultimately, EUCAIM aims to shift European imaging research from isolated, project-based data collections toward a persistent, trusted infrastructure that supports continuous, large-scale, and reproducible AI development.

Looking toward long-term sustainability, EUCAIM is designed to evolve in alignment with the EHDSR. The governance tools, contractual templates, and technical standards developed within EUCAIM serve as reusable components for future health data spaces in other domains, such as genomics, electronic health records, and wearable device data-thereby facilitating convergence toward interoperable European research infrastructures. Sustainability will depend on transitioning from project-based funding to operational financing models, potentially through integration with national health data access bodies established under the EHDSR, as well as sustained institutional commitment from participating countries and healthcare systems.

International collaboration beyond the EU regulatory context presents both opportunity and challenge. While EUCAIM principles and experience could inform similar initiatives globally, direct replication would require adaptation to local legal frameworks—such as HIPAA in the United States or PIPL in China-that differ from GDPR fundamentally in their approach to data protection. Nevertheless, EUCAIM remains fully open to participating in international projects that pursue federated, interoperable data-sharing approaches.

## Conclusion

In summary, EUCAIM represents an implementation-driven paradigm for the secure, reliable, and sustainable reuse of medical imaging data. EUCAIM demonstrates how compliance with ethical and legal standards can coexist and be enabled by technological innovation. By fostering interoperability, transparency, and trust across European Member States, it lays the foundations for a truly data-driven pan-European health research ecosystem capable of accelerating scientific discovery in medical imaging and improving patient outcomes.

## Supplementary Material

umag018_Supplementary_Data

## Data Availability

No datasets were used in this manuscript.
